# Pseudo-Dynamic Test on Composite Frame with Steel-Reinforced Recycled Concrete Columns and Steel Beams

**DOI:** 10.3390/ma15186355

**Published:** 2022-09-13

**Authors:** Jing Dong, Xiaoran Cong, Hui Ma, Yunhe Liu, Ying Wang

**Affiliations:** 1 State Key Laboratory of Eco-Hydraulics in Northwest Arid Region, Xi’an University of Technology, Xi’an 710049, China; 2 School of Water Resources and Hydro-Electric Engineering, Xi’an University of Technology, Xi’an 710049, China

**Keywords:** steel reinforced recycled concrete column and steel beam, composite frame, pseudo-dynamic test, seismic response, hysteretic energy dissipation

## Abstract

Pseudo-dynamic tests were conducted on a 2/5-scaled, three-story, two-span frame with steel-reinforced recycled concrete (SRRC) columns and steel beams. The El-Centro earthquake waves, Taft earthquake waves, and Lanzhou artificial earthquake waves were considered as the main loads to study the seismic behavior. The failure modes, displacement and acceleration time history curves, hysteretic characteristics, energy dissipation, stiffness degradation, and inter-story drift capacity of the composite frame were analyzed. Results showed that the composite frame did not show plastic deformation during the whole test process, and the steel beams and columns basically did not yield. Under the action of three seismic waves, the displacement response, acceleration response, and restoring force response of the composite frame were increased with seismic intensity, while the hysteretic curves and energy dissipation were different as the seismic wave changed. The seismic response of the composite frame was greatly affected by the spectral characteristics of the loading ground motion and the hysteretic energy mainly consisted of recoverable elastic deformation energy. Under the action of Taft wave with the input peak acceleration of 400 gal (rare earthquake), the stiffness degradation of composite frame was the largest, which reduced to 47% of the initial stiffness. This indicated that the composite frame had good energy dissipation performance in the elastic and elastic-plastic stages, and still possessed good rigidity after a rare earthquake, fully achieving the design purpose of “no collapse in major earthquake”.

## 1. Introduction

In 1987, the Brundtland Report first systematically expounded the idea of sustainable development to the world. Sustainable development was development that met the needs of the present without compromising the ability of future generations to meet their own needs. Sustainable development remains the main theme of the current world development. Green buildings and sustainable buildings are an important part of sustainable development, the aims of which include a reduced carbon footprint, strengthened level of ecology and environmental protection, improved indoor and outdoor environmental conditions, and the efficient use and conservation of resources [1,2].

With rapid urbanization in the world, concrete has become the most consumed construction material and traditional buildings have gradually developed into green buildings, passive buildings, and sustainable buildings [3,4]. At present, the assessment standard of green buildings is a general evaluation system. The evaluation standard considers building that integrate the characteristics of comfort, convenience, and sustainable development. The assessment standard of green buildings is divided into five first-class indicators, including safe and durable, healthy and comfortable, convenient life, resource conservation, livable environment. Among them, the index of resource conservation accounts for a larger scoring weight. The combination of sustainable building and recycled concrete can make the building better meet the index of resource conservation in the assessment standard of green building. This represents an effective way to solve the problem of construction waste and strengthen the resource conservation, which is in line with the resource conservation and sustainable development strategy of the world [5,6,7,8,9]. At present, recycled concrete is not only widely used in the construction field, but also in the pavement design field [10,11].

The steel-reinforced recycled concrete (SRRC) column is a kind of composite structural member, based on the excellent mechanical performance of steel-reinforced concrete column and the material characteristics of recycled concrete and steel. It is a new type of composite member composed of steel bars in the column section, assembled structural longitudinal bars and stirrups, and finally filled recycled concrete [12]. Considering the excellent mechanical performance of steel beam and the high bearing capacity and good seismic performance of SRRC column, SRRC column was combined with steel beam to form the SRRC column and steel beam composite frame structure. Similar composite frame structure systems have been studied by many researchers at home and aboard. Chung-Che Chou et al. carried out experimental research and finite element analysis on a post-tensioned reinforced concrete column-steel beam composite frame [13]. Wei Li et al. studied the seismic behavior of concrete filled steel tubular column-steel beam joints with different connection details [14]. Some other researchers studied the seismic performance of steel-reinforced concrete column and steel beam composite frame structures using finite element analysis method on the basis of experiments [15,16,17]. Results obtained by these researches for the frames are positive and encouraging. However, few researches have studied the seismic behavior of composite frames with SRRC columns and steel beams.

Accordingly, in order to investigate seismic performance of SRRC column and steel beam composite frame, a series of pseudo-dynamic tests of a 2/5-scaled, three-story, two-span SRRC column and steel beam composite frame were performed this paper. The displacement and acceleration responses, hysteretic energy dissipation characteristics, inter-layer deformation capacity, stiffness degradation, and strain response analysis of frame were examined under three different peak acceleration conditions of seismic waves. The research conclusions provide a basis for theoretical analysis to improve the seismic design method of such composite frame structures.

## 2. Experimental Program

### 2.1. Design of SRRC Frame

**Figure 1:**
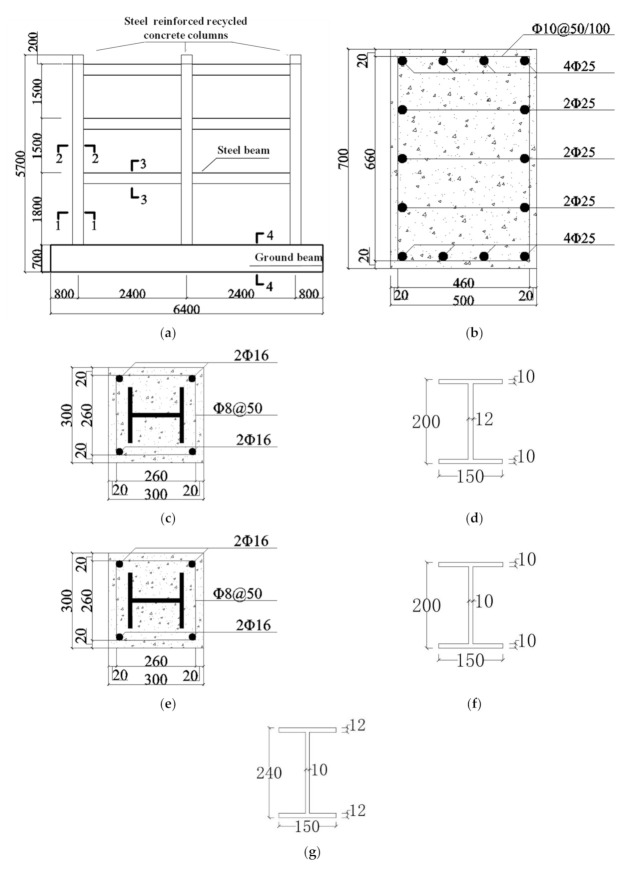
Specimen size and section details. (**a**) Specimen size; (**b**) 4-4 Cross section; (**c**) 1-1 Cross section; (**d**) 1-1 Section steel; (**e**) 2-2 Cross section; (**f**) 2-2 Section steel; (**g**) 3-3 Cross section.

The new composite frame was a 3-story, 2-span structure. The height of the bottom layer was 1800 mm, while the standard layers were 1500 mm, and both beam spans were 2400 mm. The beam and column steel of the composite frame was solid-web steel. The height of the SRRC column was 5700 mm and the section size of the SRRC column was rectangular column: 300 mm × 300 mm. The I-shaped steel of the columns and beams was welded. The section size of steel column was 140 mm × 140 mm × 10 mm × 10 mm (bottom layer), 140 mm × 140 mm × 6 mm × 8 mm (general layer), and the section size of steel beam was 200 mm × 140 mm × 8 mm × 10 mm. The concrete cover for the test specimen was 20 mm. The model size and section details are shown in Figure 1.

### 2.2. Material Performance Index

All structural steel was Q235 low carbon steel. The steel samples were retained according to the specification [18,19]. According to the tensile test of steel, the average yield strain of reinforcement was 1681 *με*, and the average yield strain of steel was 1524 *με*. The basic steel mechanical properties of different specifications were measured as shown in Table 1. A large number of experimental studies have investigated the mix proportion of recycled concrete used by our research group, which had good stability [20]. The mix ratio of recycled concrete is shown in Table 2. The mechanical properties test of recycled concrete materials was completed at the Building Materials laboratory of Xi’an University of Technology. The cube compressive strength test was measured on the universal testing machine. The average compressive strength and basic mechanical properties of the cube were measured as shown in Table 3.

### 2.3. Method and Loading Equipment

The method of pseudo-dynamic test was originally proposed by the Japanese scholar K. Takanashi who successfully completed the pseudo-dynamic structure test [21,22]. This method was mainly a seismic test research which combined testing with numerical analysis technology. Therefore, many scholars have carried out seismic simulation tests on some large-scale models or prototype structures through pseudo-dynamic test methods [23,24,25,26,27]. A pseudo-dynamic test consisted of two parts: control of loading equipment and numerical simulation of computer.

**Figure 2:**
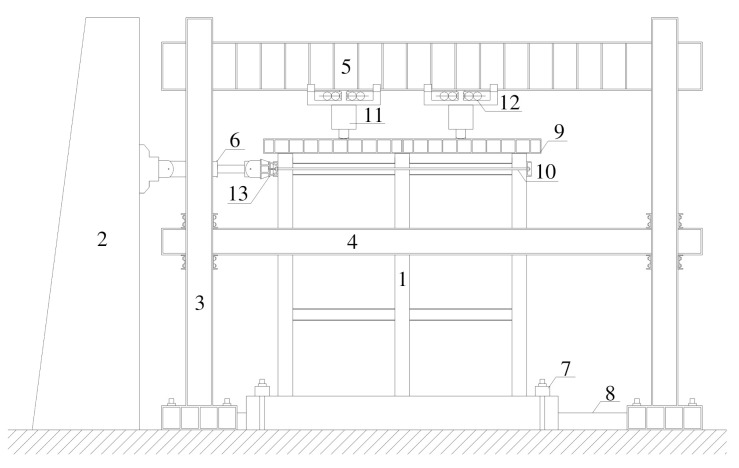
Schematic diagram of test loading device. 1. Steel reinforced recycled concrete column-steel beam composite frame specimen; 2. Reaction wall; 3. Reaction framework; 4. Lateral bracing beam; 5. Reaction girder; 6. MTS hydraulic servo actuator; 7. Pressure beam; 8. Horizontal shear embedded support; 9. Distribution beam; 10. Force transfer chain rods; 11. Oil jack; 12. Sliding support; 13. Loading end.

**Figure 3:**
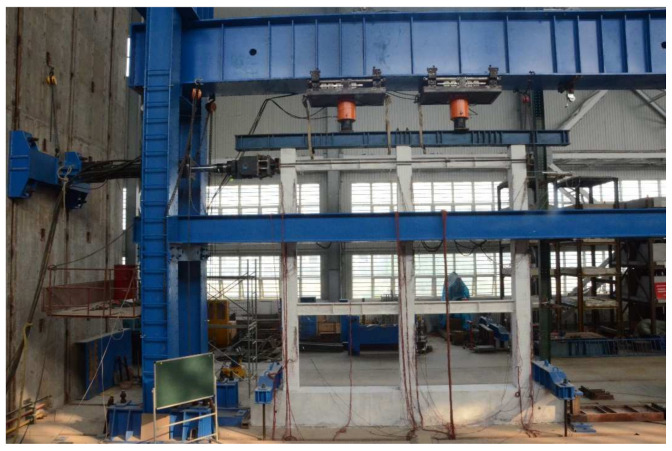
Test loading device.

The schematic diagram of the test loading device is shown in Figure 2. The vertical load of the model was applied to each column top through the top distribution beam by two jacks. The side column and the middle column were respectively subject to the axial compression ratio 0.2 and 0.4 according to the design. Then, the lateral load was applied to the elevation of the three-layer beam of the frame through MTS hydraulic servo actuator, which had a maximum lateral load of 1500 kN and a displacement capacity of ±400 mm. The test device is shown in Figure 3.

### 2.4. Test Contents and Measuring Points

**Figure 4:**
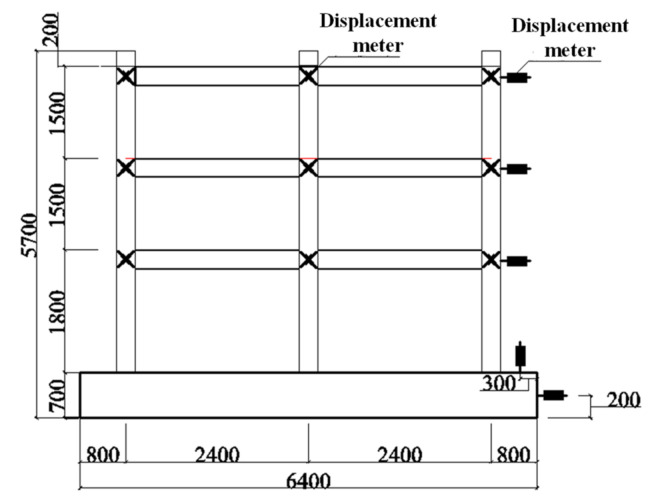
Positions of displacement meters.

**Figure 5:**
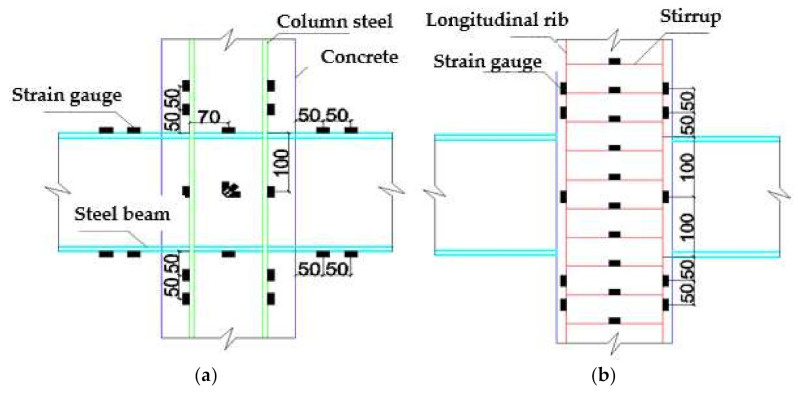
Positions of strain gauges. (**a**) Strain gauges on steel frame; (**b**) Strain gauges on steel bars.

According to the test purpose and structural characteristics of the composite frame, the main measurement contents of pseudo-dynamic test are shown as follows:

(1) Load: Lateral load was automatically collected by MTS electro-hydraulic servo system.
(2) Lateral displacement: A displacement meter was installed along the center line of each beam in the frame to measure the complete displacement of each layer. The layout of the displacement meter is shown in Figure 4.
(3) Node deformation: The two wire-drawing displacement meters were cross-layered to measure the deformation of diagonal direction in the core area of the node.
(4) Strain: The strain, including steel beam strain in plastic hinge zone at beam end, steel bar strain in plastic hinge zone at column end, stirrup strain at column end, steel web strain in joint core zone, etc., were measured. The detailed measuring points are shown in Figure 5. The data were acquired using a TDS-630 data acquisition instrument.

### 2.5. Test Loading System

**Figure 6:**
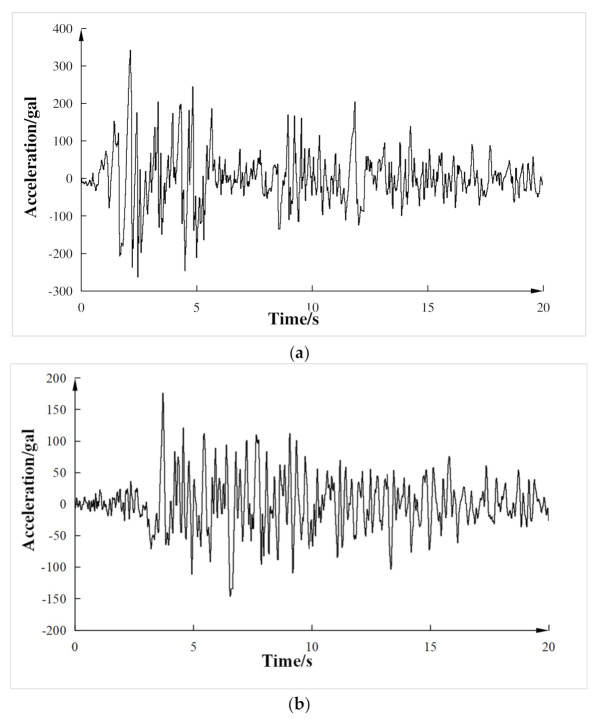

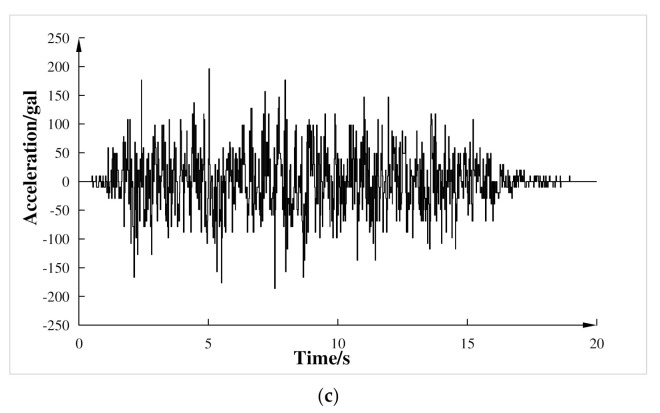
Seismic waveform. (**a**) El Centro Wave; (**b**) Taft Wave; (**c**) Lanzhou Artificial Wave.

The test loading system can directly obtain the lateral stiffness of the frame. By inputting the lateral stiffness into the loading system, the initial stiffness matrix can be calculated and obtained, and then the pseudo dynamic test force can be conducted [28]. The measured lateral stiffness of the composite frame is +20.1 kN/mm and −18.6 kN/mm, so the average value is 19.35 kN/mm, which would be inputted into the loading system. The difference between the positive and negative directions of lateral stiffness may be caused by the processing defects of the test piece, or some gaps between the loading equipment, but the error is within the allowable range and can meet the accuracy requirements. According to the seismic code, time-history analysis required at least two actual severe seismic records and artificial simulation seismic records. El Centro wave, Taft wave, and Lanzhou artificial wave were selected as input ground motions according to the test conditions. The first 20 s seismic records of three waves were taken in the pseudo-dynamic test. According to the principle of similarity coefficient, the time interval was adjusted to 0.02 s. The unit of seismic peak acceleration adopted the physical unit (gal). The SI (Système International d’Unités) stipulated that 1 gal was equal to 1 cm/s^2^. Loading conditions are shown in Table 4. Seismic waveforms are shown in Figure 6.

## 3. Test Results and Analysis

### 3.1. Test Phenomena

**Figure 7:**
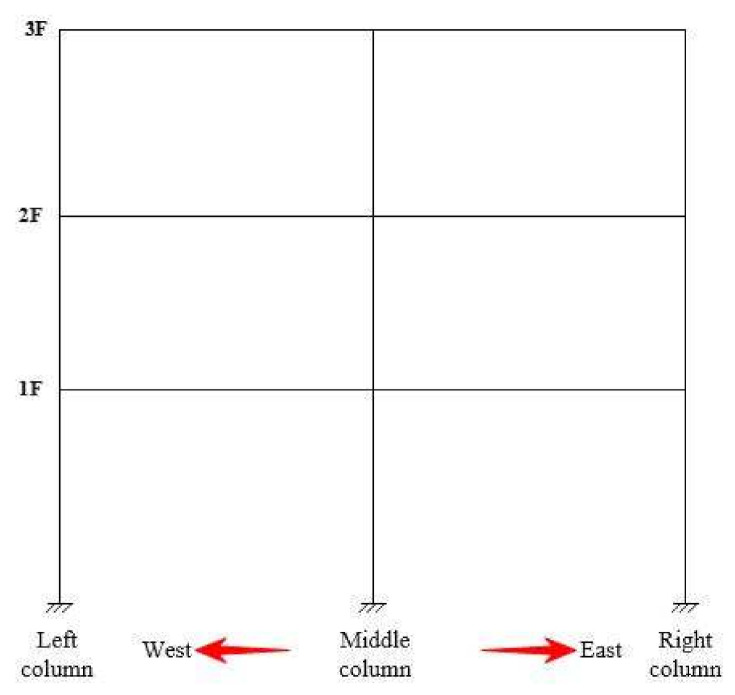
Phenomenon description diagram.

The orientation diagram of the test frame phenomenon description is shown in Figure 7. At the beginning of test, when the composite frame was subjected to El Centro, Taft, and Lanzhou artificial waves whose input peak acceleration is 35 gal, 55 gal, 70 gal, and 100 gal, the dynamic response of the composite frame was minute, the deformation was not notable, and the test specimen was basically in the elastic stage. When the peak acceleration was 200 gal, the dynamic response of El Centro wave was still the largest compared with those of Taft wave and Lanzhou artificial wave. After Taft seismic wave input with peak acceleration of 300 gal, the east-west lateral movement of the composite frame specimen was more obvious, and a small amount of cover recycled concrete spalled off. Under the action of Taft seismic wave with input peak acceleration of 400 gal, the east-west lateral movement of composite frame specimens was more notable, and the phenomenon of recycled concrete skin spalled was intensified.

**Figure 8:**
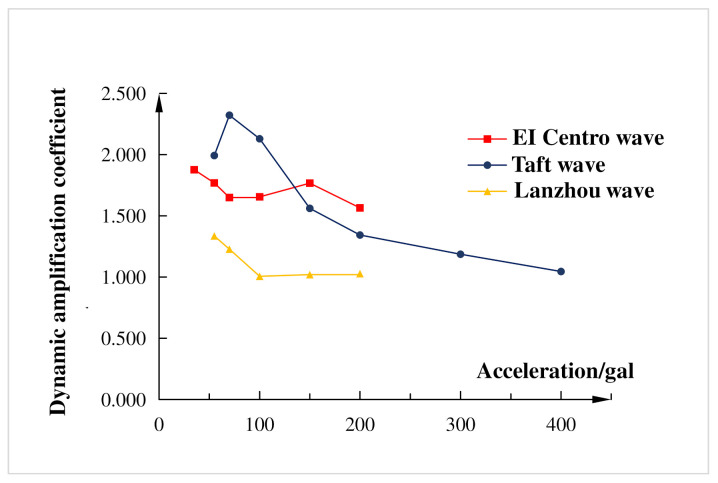
Relationship between dynamic amplification coefficient and input seismic waves.

Figure 8 shows the relationship between the dynamic amplification coefficient and the input seismic wave. The dynamic amplification coefficient was the ratio of the values of the structural response acceleration to the input seismic waves acceleration [28]. It can be seen from Figure 8 that the overall trend of the dynamic amplification coefficient of the experimental composite frame structure decreases with the increase of the peak value of the seismic wave acceleration. Under the seismic wave action of El Centro wave, Taft wave, and Lanzhou artificial wave, the average dynamic amplification coefficient of the composite frame was 1.526, and the maximum value was 2.321, which were less than the limit values of Chinese Code for Seismic Design of Buildings (GB50011-2010). This showed that the seismic performance of the composite frame was fine and met the requirements for fortification against earthquakes.

### 3.2. Displacement Response

**Figure 9:**
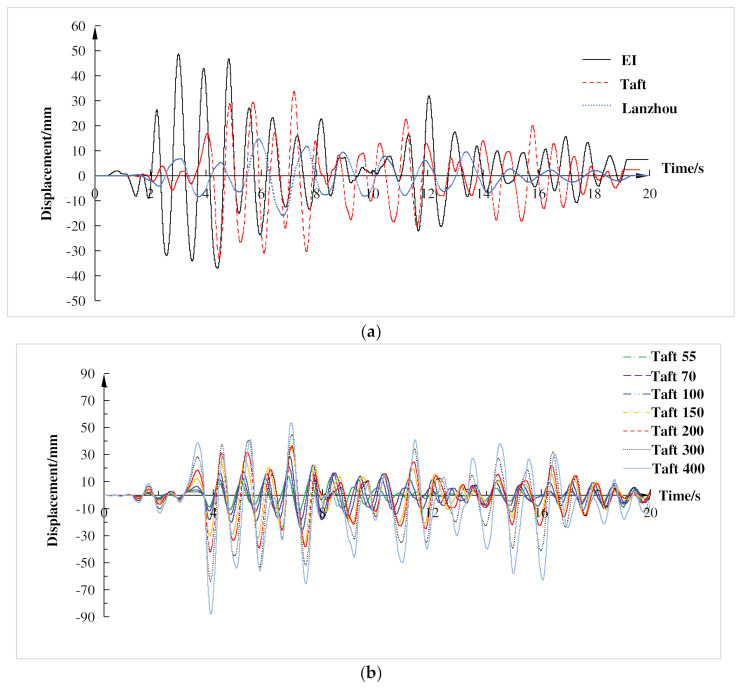
Time history curves of top displacement. (**a**) Input peak acceleration is 200 gal; (**b**) Taft wave.

Figure 9 exhibits the displacement time-history curves of the top layer of the composite frame under various working conditions. It can be seen from Figure 9 that subjected to different seismic waves, there was a time lag between the displacement response of the composite frame and the acceleration of the seismic waves, and the time of the peak value was also different. The reason for this was that the spectrum characteristics of input seismic waves and the natural frequencies of frame had a certain influence on the displacement response of frame. Only when the current natural frequencies of frame were closed to the spectrum of input waves will the displacement response reach the peak value. Under the same seismic waves, with the increase of input peak acceleration, the displacement response gradually became larger, the cumulative damage and stiffness of the composite frame changed, so the maximum displacement response value of the top layer also changed significantly. In the elastic stage, the peak displacement time of the composite frame was roughly the same. While the strain value of the steel reached the yield strength, it was determined that it was in the elastic-plastic stage. When the composite frame entered the elastic-plastic stage, the peak displacement time changed due to the stiffness degradation of the composite frame.

### 3.3. Acceleration Response

**Figure 10:**
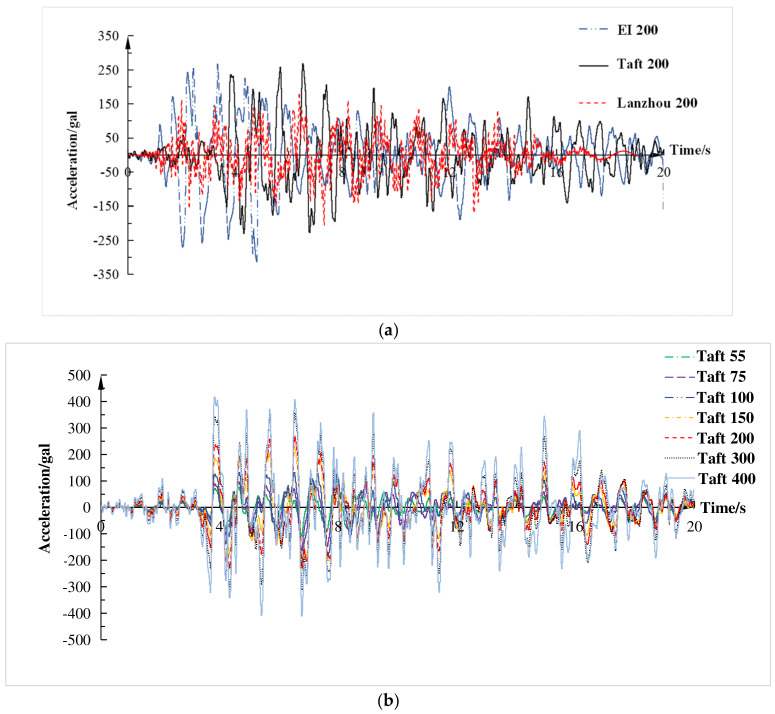
Time-history curve of top acceleration. (**a**) Input peak acceleration is 200 gal; (**b**) Taft wave.

Acceleration responses of the top layer of the composite frame under various loading conditions are shown in Figure 10. It can be seen from Figure 10 that under the action of different seismic waves, the top-level acceleration response time-history curves of the composite frame had a good agreement with that of its corresponding input seismic waves basically but did not reach the peak at the same time point. When the input peak acceleration was 200 gal, the peak acceleration response of El Centro wave, Taft wave and Lanzhou artificial wave occurred in 4.82 s, 6.54 s, 7.33 s respectively. The peak acceleration response of Taft wave appeared in the positive direction, while the other waves appeared in the negative direction. It can be seen from Figure 10 that under the action of the same seismic wave, the shapes of the top-level acceleration response time-history curves of the composite frame were roughly consistent. When the composite frame was in the elastic stage, the occurrence moments of the top-level peak values of acceleration response were the same. However, with the increase of the peak acceleration of the input seismic waves, the local part of the composite frame gradually reached the yield state, which resulted in the structure entering the elastic-plastic stage and the occurrence moments of peak values moving slightly backward. The main reason for this was that the stiffness of the frame changed, thereby affecting the natural frequency, resulting in the lag of occurrence moments of the top-level peak values of the acceleration response.

**Figure 11:**
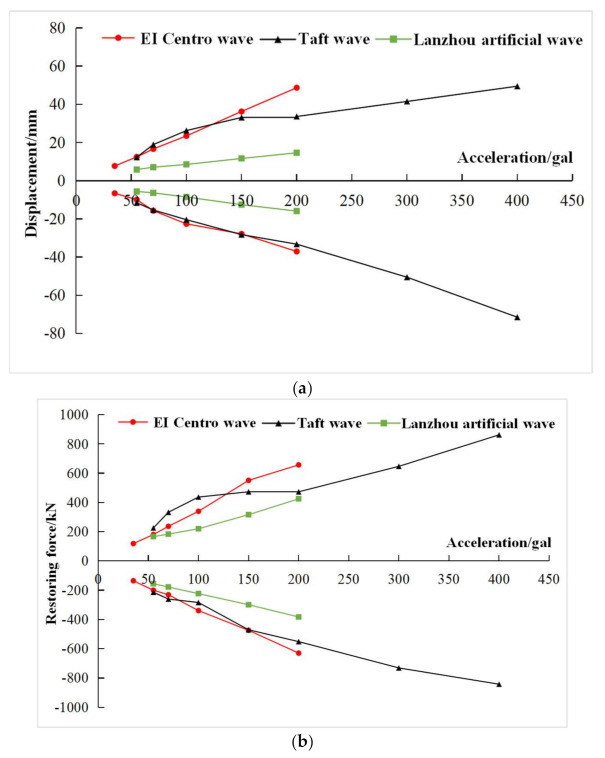
Peak acceleration-displacement curve and peak acceleration-restoring force curve.; (**a**) Peak acceleration-displacement curve; (**b**) Peak acceleration-restoring force curve.

The peak acceleration–displacement curves and peak acceleration-restoring force curves are shown in Figure 11. As shown in Figure 11a, under the action of seismic waves, the displacement was basically linear growth with the increase of peak acceleration, indicating that the stiffness of the structure was uniformly distributed and there was no obvious weak part. It can be seen from Figure 11b that the restoring force was also direct proportional to the input peak acceleration. When the input peak acceleration was the same, the peak displacement and restoring force of El Centro wave and Taft wave were relatively close, while the Lanzhou artificial wave was relatively small, which was corresponding to the spectral characteristics of the three waves.

### 3.4. Hysteretic Characteristics

**Figure 12:**
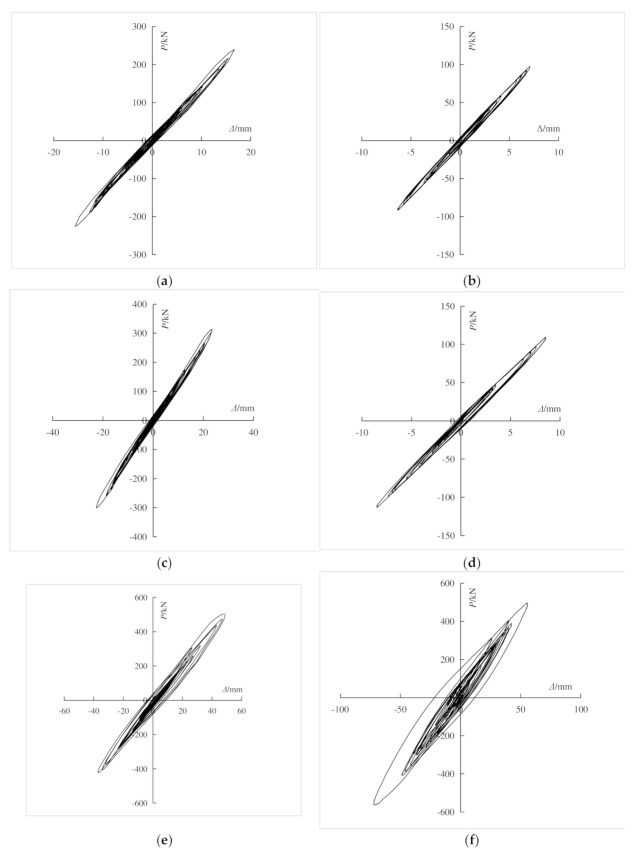
Hysteretic curve of base shear-top displacement. (**a**) 70 gal El Centro wave; (**b**) 70 gal Lanzhou wave; (**c**) 100 gal El Centro wave; (**d**) 100 gal Lanzhou wave; (**e**) 200 gal El Centro wave; (**f**) 400 gal Lanzhou wave.

According to the pseudo-dynamic test data of the composite frame, the base shear-top displacement hysteretic curves of the composite frame were obtained under each working condition. As shown in Figure 12, with the same peak acceleration, three seismic waves had different effects on the hysteretic curves and energy dissipation of the composite frame. Among the dynamic response of three waves, the El Centro wave was the largest and the Lanzhou artificial wave was the smallest. With the increase of the input peak acceleration, the enveloped area of each hysteretic loops increased, and the energy dissipation of the structure increased. Under the action of Taft wave with peak acceleration of 400 gal, only the composite frame showed cracks, and only a few parts enter the yield state, which showed that the composite frame can maintain good integrity and had good seismic capacity under strong earthquakes.

### 3.5. Energy Dissipation

In the pseudo-dynamic test, since the influence of velocity on the structure was slight such that damping energy dissipation could be ignored, the influence of hysteretic energy dissipation could be mainly considered [26]. Hysteretic energy dissipation was the main form of total input seismic energy, which mainly referred to the ability of plastic deformation and energy absorption of structures or components under seismic action. The cumulative hysteretic energy dissipation was calculated as follows [29]:

(1) $$E_{h}=\sum_{i=0}^{n}\frac{1}{2}\left(F_{i+1}+F_{i}\right)\left(X_{i+1}+X_{i}\right)$$

where *F*_i + 1_ and *F*_i_ are the restoring force of (*i* + 1)^th^ point and *i*^th^ point, respectively; *X*_i + 1_ and *X*_i_ are the displacements corresponding to restoring force.

**Figure 13:**
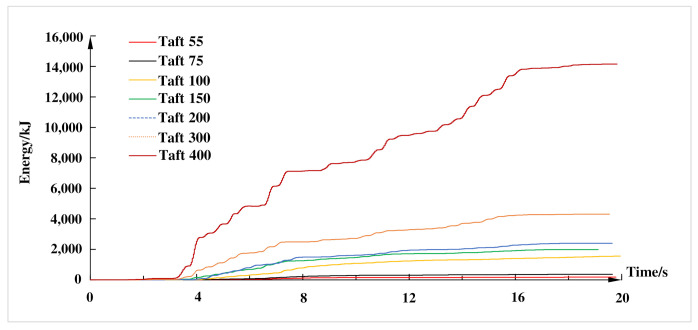
Hysteretic energy dissipation capacity curves of structure.

The energy dissipation time-history of the composite frame under different working conditions of Taft wave are shown in Figure 13. It can be seen from Figure 13 that when the peak acceleration of input seismic waves was small, the cumulative hysteretic energy dissipation of the composite frame was also small. When the composite frame was gradually changed from elastic stage to elastic-plastic stage, the hysteretic energy dissipation of the frame was significantly improved. With the increase of the input peak acceleration, the energy consumption of the composite frame increased sharply, and the energy dissipation was nonlinear growth with the increase of the input seismic waves. This further showed that when the peak acceleration was small, the composite frame was basically in elastic state, and the hysteretic energy mainly consisted of recoverable elastic strain energy. When the frame changed from elasticity to plasticity, the hysteretic energy dissipation of the frame will increase sharply. The experimental results showed that the crack development on the composite frame was not obvious at this time, which can show that the composite frame has a high safety reserve.

**Figure 14:**
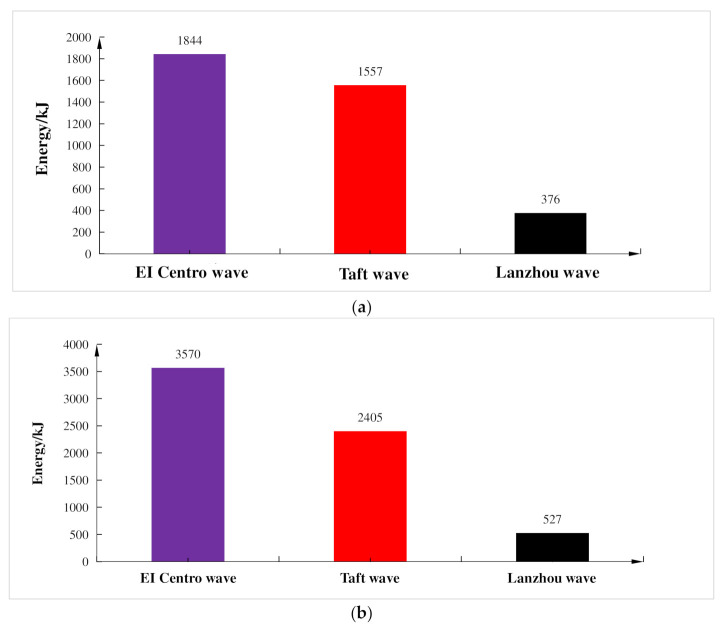
Hysteretic energy dissipation capacity curves of structure. (**a**) Input peak acceleration is 100 gal; (**b**) Input peak acceleration is 200 gal.

Energy dissipation of the composite frame under different seismic waves and different working conditions is shown in Figure 14, illustrating that when the input peak acceleration was the same, the influence of different seismic waves on the energy dissipation of the composite frame was different. Among the three seismic waves affecting energy dissipation, the influence of El Centro wave was largest, and the Lanzhou artificial wave was smallest, which showed that the seismic response of the composite frame was greatly affected by the spectral characteristics of each seismic wave.

### 3.6. Strength and Stiffness

**Figure 15:**
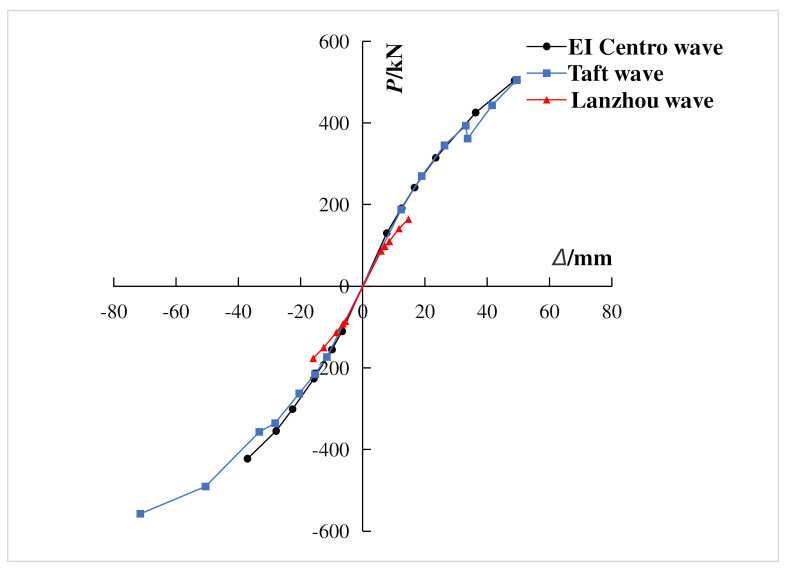
Skeleton curve of composite frame.

Figure 15 is the skeleton curve of shear-top displacement. It was found that the base shear of the composite frame increased with a large deformation. When the input peak acceleration reached 400 gal, the inter-story drift ratio of the composite frame was 1/77. However, at this time, the skeleton curve did not present a downward section, and there was still an upward trend, which fully showed that the composite frame had strong lateral resistance, large deformation capacity, and high bearing capacity.

**Figure 16:**
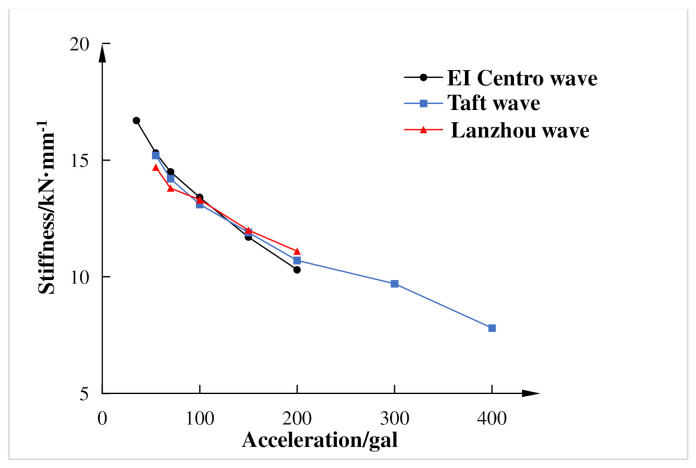
Stiffness degradation curve of model structure.

The stiffness of the composite frame was mainly the secant stiffness of the maximum restoring force point. Figure 16 shows the stiffness variation diagram. It can be seen from the figure that under the same input peak acceleration of El Centro wave, Taft wave, and Lanzhou artificial wave, the stiffness degradation of the composite frame was basically consistent, which indicated that the spectral characteristics of seismic waves had little effect on the stiffness degradation performance of the composite frame, and the main influencing factor was the peak acceleration of seismic waves. With the input acceleration and input seismic energy increased, the damages of the composite frame under the action of seismic waves were inevitably accumulated, and the stiffness of the frame was also decreased. Finally, the basic period of the frame became larger, and the natural vibration frequency was gradually reduced. This showed that the composite frame had good energy dissipation performance in elastic and elastic-plastic stages and still had good rigidity after a rare earthquake.

### 3.7. Inter-Story Drift Capacity

**Figure 17:**
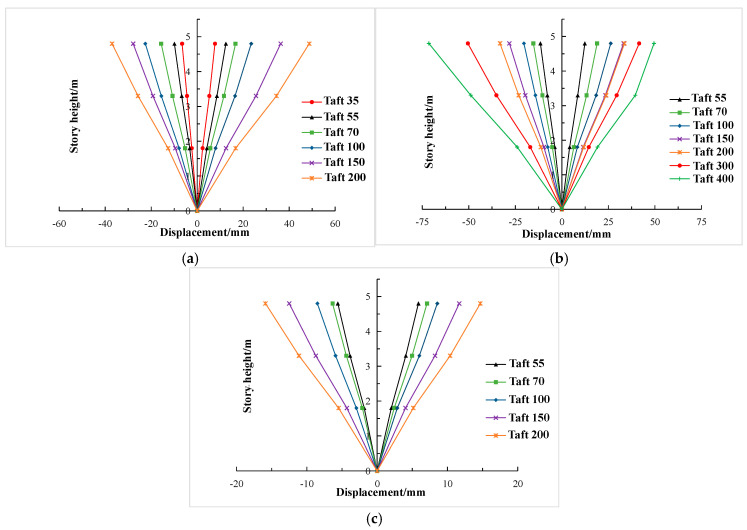
Envelope diagram of displacement response along height. (**a**) El Centro wave; (**b**) Taft wave; (**c**) Lanzhou artificial wave.

According to the displacement data obtained from the pseudo-dynamic test, the envelope diagram of displacement response of three seismic waves along the height was drawn, as shown in Figure 17. The envelope diagram can visually reflect the change trend of displacement during the loading process. It can be seen from Figure 17 that under the action of each seismic wave, with the increase of input peak acceleration, the displacement of the top layer was obviously larger compared with those of the bottom and the second layer. The displacement distribution of each layer was basically an inverted triangle, which showed that the displacement response of each layer was relatively uniform, and also reflected that the stiffness of the composite frame was uniformly distributed from top to bottom. The displacement of each layer of the composite frame was symmetrical in positive and negative directions, which indicated that the lateral stiffness of the composite frame in positive and negative directions was also symmetrical, further indicating that the integrity of the frame was good.

### 3.8. Strain Response Analysis

**Figure 18:**
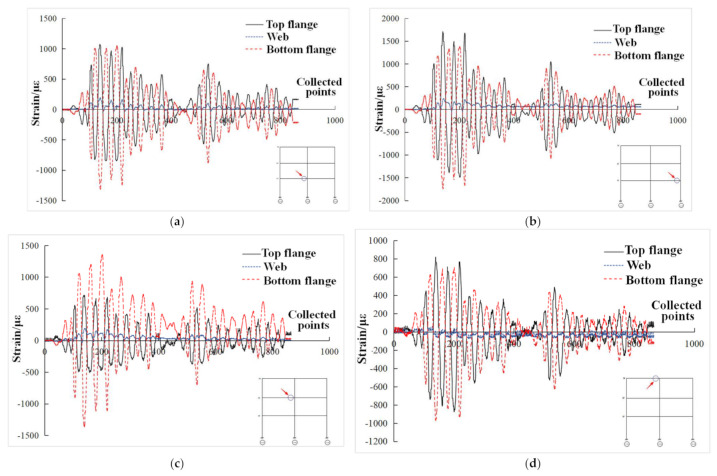
Strain of beam end. (**a**) Strain at left beam end of middle node in first layer; (**b**) Strain at beam end of one layer of right side joint; (**c**) Strain at the left beam end of the middle node in second layer; (**d**) Strain at left beam end of third layer middle joint.

**Figure 19:**
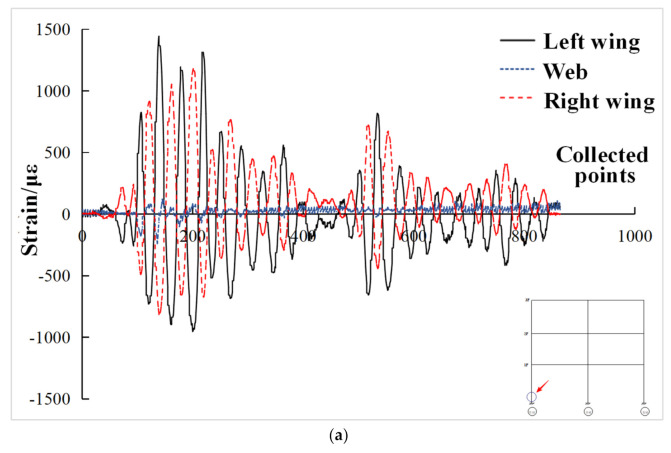

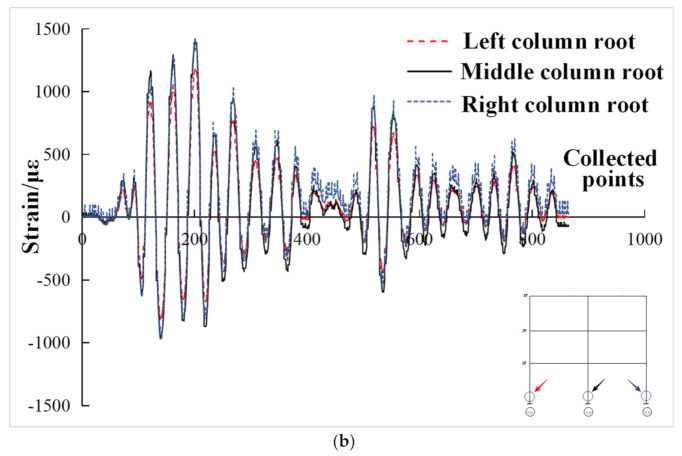
Strain at the root of column steel in composite frame. (**a**) Strain at the root of left steel column; (**b**) Strain at the root of each steel column.

Under the action of El Centro wave with input peak acceleration of 200 gal, the strain curve of beam end of each layer of composite frame structure is shown in Figure 18. It can be seen from the figure that the strain values of the web plate at the beam end were much smaller than that of the upper and lower flanges, which was consistent with the mechanical characteristics of I-steel. The web plate mainly bore shear stress, while the flange mainly bore positive stress. It can also be seen from the figure that the strains of the upper and lower flanges were basically symmetrically distributed, and the strain values at the symmetrical positions of the composite frame were basically consistent and symmetrically distributed. Under the action of El Centro wave with input peak acceleration of 200 gal, the strain curve of the steel column root of each column of the composite frame structure is shown in Figure 19. It can be seen from Figure 19a that the strain of column steel was similar to that of beam end, and the strain of the flange was symmetrical. The strain values of the flange were much larger than that of the web. Each strain value did not reach the yield strain of the steel and was still in the elastic stage. From Figure 19b, it can be seen that the strain change trend of column steel in the root of the column was basically consistent, which reached the yield strain value. It can be stated that the integrity of the composite frame was good and the stress of each column was basically equivalent.

**Figure 20:**
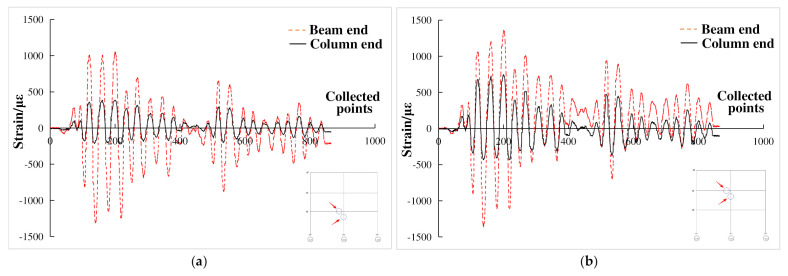

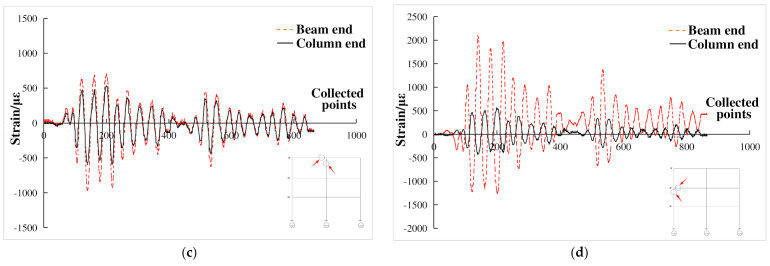
Strain at beam end and column end of composite frame. (**a**) Nodes in the first layer; (**b**) Nodes in the second layer; (**c**) Nodes in the third layer; (**d**) The left side node of the second layer.

Under the action of El Centro wave with input peak acceleration of 200 gal, the strain curve of beam end and column end at each node of the composite frame structure is shown in Figure 20. It can be seen from the diagram that the strain variation law of beam end and column end was basically consistent, but the strain value of beam end was much larger than that of column end. The maximum strain of column end was about 500 *με*, which was obviously in the elastic stage. The maximum strain of beam end was above 1000 *με*, and the peak strain of beam end of the edge node reached 2100 *με*, which satisfied the principle of “strong column weak beam” in the seismic design of structures.

**Figure 21:**
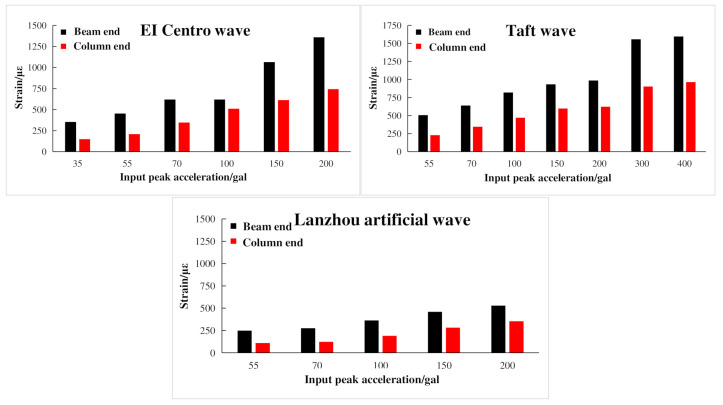
Peak strain at beam end and column end of composite frame.

Under the action of El Centro wave, Taft wave, and Lanzhou artificial wave, the peak strain of mid-story beam and column steel in the composite frame structure is shown in Figure 21. It was found that when the input peak acceleration continuously increased, the strain values at the beam end and column end were also increased, but the strain at the column end had been in elastic strain. At the same time, comparing the influence of three seismic waves on the peak strain of beam end and column end, the descending order was Taft wave, El Centro wave, and Lanzhou artificial wave.

## 4. Discussion

In order to discuss the seismic performance of the SRRC column-steel beam composite frame, the SRRC column-steel beam composite frame was compared with steel-concrete composite column frame, recycled concrete-encased steel frame, concrete-filled steel tubular columns composite frame with buckling brace, steel-concrete composite joint frame, and steel structure frame [23,24,25,26,29]. In the pseudo-dynamic test, under the action of seismic wave, the failure stage and failure mode of SRRC column-steel beam composite frame were roughly the same as those of steel-concrete composite structure. The displacement response, acceleration response, and restoring force response of the composite frame were increased with the increase of seismic intensity, but there was time lag between the peak displacement response and the peak restoring force. The average dynamic amplification coefficient of the SRRC column-steel beam composite frame was 1.526, and the maximum value was 2.321. The value was higher than the average dynamic amplification coefficient of the steel-concrete composite column frame and less than the average dynamic amplification coefficient of the steel-concrete composite joint frame. Through analyzing the hysteretic curve and hysteretic energy dissipation time history curve of the composite frame, the energy dissipation capacity of the SRRC column-steel beam composite frame was different from that of concrete-filled steel tubular columns composite frame with buckling brace, recycled concrete-encased steel frame, steel-concrete composite column frame, and steel structure frame. Compared with the concrete-filled steel tubular columns composite frame with buckling brace and the steel structure frame, the SRRC column-steel beam composite frame still showed room for improvement, while the SRRC column-steel beam composite frame proved able to fully meet the seismic fortification requirements of the structure in the test. Furthermore, the use of recycled concrete is the main theme of sustainable development in the current world. With further research on the application of recycled concrete and a reasonable design, the SRRC column-steel beam composite frame may have a broader application prospect.

## 5. Conclusions

Based on the pseudo-dynamic test of the composite frame, the hysteretic characteristics, energy dissipation, strength and stiffness, inter-story deformation capacity, and other dynamic characteristics of the composite frame were obtained and analyzed. The main conclusions are summarized as follows:

(1) Under the action of different input peak accelerations of three seismic waves, the displacement response, acceleration response, and restoring force response of the composite frame were increased with seismic intensity, but there was time lag between the peak displacement response and the peak restoring force.
(2) Under the action of different seismic waves with the same peak acceleration, the seismic response of the structure was greatly affected by the spectral characteristics of the loading ground motion. When the input peak acceleration of seismic was small, the cumulative hysteretic energy dissipation of the composite frame was also small, which showed that the hysteretic energy mainly consisted of recoverable elastic deformation energy.
(3) Under the action of Taft wave with the input peak acceleration of 400 gal, the stiffness degradation of the composite frame was the largest, which reduced to 47% of the initial stiffness. This showed that the composite frame had good energy dissipation performance in elastic and elastic-plastic stages, and still had good rigidity after a rare earthquake.
(4) From the above findings and analysis, the composite frame possesses excellent seismic performance, and all indicators could meet the requirements of the code in the laboratory well. The research and application of recycled concrete is in line with the theme of green environmental protection and ecologically sustainable development all over the world. Moreover, the application of the above frame would make a small contribution to the treatment of construction waste.

## Figures and Tables

**Table 1:** Mechanical performance indexes of steel.

Steel Diameter (Thickness)/mm	Yield Strength /MPa	Ultimate Strength /MPa	Yield Strain /10^−6^	Elastic Modulus /MPa
Reinforcement diameter				
8	391.83	555.79	1771	221.2 × 10^3^
10	361.23	515.43	1643	219.9 × 10^3^
16	377.48	531.7	1776	212.5 × 10^3^
25	306.12	398.16	1534	199.6 × 10^3^
Steel plate thickness				
6	300.51	452.28	1485	202.3 × 10^3^
10	280.27	407.48	1568	212.1 × 10^3^
12	293.91	415.78	1519	193.5 × 10^3^

**Table 2:** Mix ratio of recycled concrete.

Recycled Concrete Strength Grade	Replacement Rate	Cement-Water Ratio (kg/m^3^)	Cement (kg/m^3^)	Sand (kg/m^3^)	Natural Coarse Aggregate (kg/m^3^)	Recycled Coarse Aggregate (kg/m^3^)	Water (kg/m^3^)
C40	100%	0.43	464	585	0	1187	195

**Table 3:** Basic mechanical properties of recycled concrete.

Strength Grade	Replacement Rate/*r*	Cube Compressive Strength *f*_rcu_/MPa	Axial Tensile Strength *f*_rc_/MPa	Tensile Strength *f*_rt_/MPa	Elastic Modulus *E*_rc_/MPa
C45	100%	51.8	39.4	12.4	2.79 × 10^4^

Note: *f*_rcu_ was cube compressive strength of recycled concrete, *f*_rc_ was prism axial compressive strength of recycled concrete, *f*_rt_ was tensile strength of recycled concrete, *E*_rc_ was elastic modulus of recycled concrete, *f*_rc_ = 0.76 *f*_rcu_; *f*_rt_ = 0.24 *f*_rcu_; *E*_rc_ = 10^5^/(2.8 + 40.1/*f_rcu_*).

**Table 4:** Pseudo-dynamic loading conditions.

Loading Conditions	Seismic Waves	Peak Acceleration/Gal	Loading Conditions	Seismic Waves	Peak Acceleration/Gal
1	El Centro	35	10	Taft	150
2	El Centro	55	11	Taft	200
3	El Centro	70	12	Taft	300
4	El Centro	100	13	Taft	400
5	El Centro	150	14	Lanzhou	55
6	El Centro	200	15	Lanzhou	70
7	Taft	55	16	Lanzhou	100
8	Taft	70	17	Lanzhou	150
9	Taft	100	18	Lanzhou	200

## Data Availability

Not applicable.
